# Personas for the translational workforce

**DOI:** 10.1017/cts.2020.2

**Published:** 2020-01-10

**Authors:** Sara Gonzales, Lisa O’Keefe, Karen Gutzman, Guillaume Viger, Annie B. Wescott, Bailey Farrow, Allison P. Heath, Meen Chul Kim, Deanne Taylor, Robin Champieux, Po-Yin Yen, Kristi Holmes

**Affiliations:** 1 Galter Health Sciences Library and Learning Center, Northwestern University Feinberg School of Medicine, Chicago, IL, USA; 2 Center for Data-Driven Discovery in Biomedicine, The Children’s Hospital of Philadelphia, Philadelphia, PA, USA; 3 Department of Pediatrics, Perelman School of Medicine at the University of Pennsylvania, Philadelphia, PA, USA; 4 Department of Biomedical and Health Informatics, The Children’s Hospital of Philadelphia, Philadelphia, PA, USA; 5 The Oregon Health & Science University Library, Oregon Health & Science University, Portland, OR, USA; 6 Institute for Informatics, Department of Medicine, Division of General Medical Sciences, Washington University School of Medicine, St. Louis, MO, USA; 7 Goldfarb School of Nursing, Barnes-Jewish College, BJC HealthCare, St. Louis, MO, USA; 8 Department of Preventive Medicine, Division of Health and Biomedical Informatics, Northwestern University Feinberg School of Medicine, Chicago, IL, USA

**Keywords:** Methods and processes, translational workforce, implementation, interprofessional team, technology, patient perspectives, persona, qualitative, user-centred design, organisational and social issues

## Abstract

Twelve evidence-based profiles of roles across the translational workforce and two patients were made available through clinical and translational science (CTS) Personas, a project of the Clinical and Translational Science Awards (CTSA) Program National Center for Data to Health (CD2H). The persona profiles were designed and researched to demonstrate the key responsibilities, motivators, goals, software use, pain points, and professional development needs of those working across the spectrum of translation, from basic science to clinical research to public health. The project’s goal was to provide reliable documents that could be used to inform CTSA software development projects, educational resources, and communication initiatives. This paper presents the initiative to create personas for the translational workforce, including the methodology, engagement strategy, and lessons learned. Challenges faced and successes achieved by the project may serve as a roadmap for others searching for best practices in the creation of Persona profiles.

## Introduction

The Clinical and Translational Science Awards (CTSA) Program National Center for Data to Health (CD2H, Grant U24TR002306) was established in 2017 to accelerate advancements in informatics by promoting data reuse and interoperability, tool sharing, informatics fluency, and collaboration across the CTSA community [1]. Assessing and addressing the translational science community’s needs related to data, informatics, and tools has been a prime focus of the CD2H, and one that is of increasing importance as data and informatics play an ever-increasing role in research and patient care. At the heart of this are the needs and motivations of individuals employed across the spectrum of clinical and translational science (CTS), the translational workforce. The translational sciences community as well as the National Center for Advancing Translational Sciences describes translational science as spanning Basic Science Research to Pre-Clinical Research and Clinical Research, to Clinical Implementation, and ultimately on to improved Public Health—acknowledging that translational science in practice is a complex orchestration of interactions, collaborations, innovations, and implementations moving within and across all phases. This complex, yet critical process underscores the need to assess individual needs and motivations across these key signposts along translation. A six-month-long project to create *persona profiles* was proposed as a method to uncover the needs, motivations, goals, and pain points of employees in CTS.

Since the 2004 publication of Alan Cooper’s *The Inmates are Running the Asylum: Why High-Tech products Drive us Crazy and how to Restore the Sanity*, professionals in the software development world have been familiar with the concept of the persona. According to Usability.gov, a Federal resource focused on improving the user’s experience with software tools, “the purpose of personas is to create reliable and realistic representations of your key audience segments for reference.” [2] In public sector software development projects, personas are often employed by teams of programmers and project managers, to identify the various audience members who will use a software tool, to research and identify their needs and goals both in general and with regard to the tool, and to generate persona “avatars” or representations of these audience segments to provide guidance in the development process. Though based on the needs and experiences of real people, the persona profiles are not descriptions of particular real individuals, but rather fictionalised syntheses of the needs of multiple people working in the same role. The benefits that persona profiles bring to software development teams include elucidating how various user groups will utilise a tool, how users might expect a software tool to look or behave, and what types of written content users may expect from a website. Though not hard coded into any systems themselves, the persona profiles serve as a guide and reference whenever the software teams need to know how real-world users will interact with their tools. In essence, the goal of personas is to enable effective and efficient user-centred design.

The popularity of personas has grown beyond the realm of user experience (UX) design in recent years, as they have increasingly been adopted by medical service providers as a means of understanding client bases. For example, personas have been used to examine and represent the needs and behaviors of patients through several projects including Voices of Veterans and Northwestern Digital Personas [3, unpublished data]. However, until the proposed CD2H project, no concerted effort had been made to enumerate and describe roles in the clinical and translational workforce in a persona style. The resulting project, CTS Personas, was envisioned to fill this gap.

## Background: Why Are Personas Needed?

In order to design services to meet the needs of the translational workforce, those needs must be understood to the greatest extent possible. Personas can provide that understanding. As indicated by Cynthia LeRouge et al., “user profiles and personas go well beyond demographics, as they attempt to ‘capture’ the user’s mental model comprising of their expectations, prior experience and anticipated behavior … how they think, feel, and behave.” [4] To achieve an understanding of the mental models shared by groups of people working in specific positions in translational science, research into behaviors, thoughts, and feelings about their roles is crucial. While responsibilities and day-to-day activities can be gleaned from articles, websites, and job descriptions, the feelings and motivations behind CTS employees’ actions can best be approached through one-on-one conversations with people currently employed in CTS roles.

Faced with the seemingly time-consuming task of conducting research and interviews to inform persona profiles, members of the CTS workforce may question why profile documents describing their colleagues are needed at all. While working shoulder-to-shoulder with colleagues on translational projects, members of the CTS workforce may feel that they understand much about the responsibilities and concerns of the physician scientists, clinical research coordinators (CRCs), data analysts, developers, basic scientists, and biostatisticians with whom they collaborate on team projects.

A peek inside the goals, motivators, and pain points of CTS employees, however, can reveal some of the behind-the-scenes facets of their everyday lives that have a large impact on the effectiveness of clinical trials and by extension, translational research. For instance, around 40–55% of a research administrator’s (RA) time in clinical trial activation is spent on contract and budget negotiation, which can mean as much as 44 days within a 70–80 day activation process [5]. By understanding the burden that contract and budget negotiation place on an RA, CTSA sites may be inspired to examine ways in which such processes can be streamlined, such as through the use of standardised or pre-negotiated contract and budget forms. Similarly, many CRC struggle with heavy project demands, often ill-defined boundaries on their duties, and doubts about their level of understanding of clinical research, which may impede them from speaking up on matters of analysis or study design. Professionalisation through obtaining a CRC certification or through membership in CRC professional development groups may go a long way towards addressing such concerns [6]. Such professional development needs could be met by individual CTSA sites or through the national CTSA network.

Behind-the-scenes motivations for investigators are equally compelling. Researchers are becoming increasingly interested in interdisciplinary work [7] in order to facilitate translation of discoveries from bench to bedside; however, interdisciplinary work faces an important stumbling block when institutional reward systems mainly benefit single-discipline, “siloed” projects. While some institutions have begun to reward interdisciplinary work in their hiring and promotion practices, as many as two-thirds of CTSAs have not yet seriously begin to consider such updates to their practices [8].

These are but a few examples of the concerns and motivations of CTS employees that persona profiles can help to elucidate. The driving goal of the CD2H Personas project was to build a more comprehensive organised library of such goals and concerns and to share it broadly with the CTSA Program, given the lack of existing resources focused on translational workforce concerns and motivations.

### CD2H Personas Deliverables

As a Phase II project of the CD2H, with a timeline spanning January–September 2019, the CTS Personas project was conceived with a clear timeframe and list of deliverables. One dozen roles were to be profiled in one-page documents designed for easy distribution as both downloads and paper flyers. All associated articles and internet resources used to generate the profiles were to be aggregated in a bibliography. A guidebook to orient new users to the Persona profiles was to be created, as well as an onboarding video and webinar. All deliverables and related project materials were to be made openly available under an Attribution 4.0 International (CC BY 4.0). This international permissive license allows users to copy and redistribute the material in any medium or format as well as adapt the materials through remixing, transforming, and building upon the material for any purpose, even commercially, extending the downstream value of the persona profiles.

## Methods

Beginning in January 2019, a project team led by Northwestern University and including data engineers and informatics professionals from Washington University, Children’s Hospital of Philadelphia, and Oregon Health & Science University met to outline a project schedule and methodology. As part of the larger Center for Data to Health standard operating procedure, project management took place in GitHub [9], and the collaborative and open project documents were shared through Google Docs. Milestones in the project were defined as follows: (1) identify key roles to profile in the CTS landscape, (2) select elements for the Persona profile template, (3) inform the profiles from literature searches, (4) inform the profiles from interviews, and (5) construct the Persona profiles and user guidebook.

### Step 1: Identify Key CTS Roles for Profiles

To obtain the most thorough picture possible of CTS roles, the Personas team examined staff lists and organisational charts available on the CTSA hubs’ websites, tracking each resource we discovered in a Google Sheet [10]. For the 79 unique positions identified, the team obtained at least two and often three job postings for the position. CTS-Personas team member and Data Engineer Meen Chul Kim of the Children’s Hospital of Philadelphia performed various text-mining and graph analyses of the text of the job descriptions, including term frequency measures and topic modeling, as well as a cluster graph of term co-occurrence by job category and title. The cluster graph analysis proved most useful as it resulted in distinct nodes which were analysed for the most frequently occurring terms. Terms common to all of the position descriptions were thrown out (experience, work, program, position, application, skill, ability, staff, service, and health), and the most frequently occurring terms that were not common to all the job descriptions were retained (development, project, data, study, team, system, clinical, care, patient, and education). Qualitative procedures followed this work, as the 79 jobs identified were organised into 10 clusters based on these anchor terms. The team members then voted on the jobs they believed were most critical to represent in the inaugural round of one dozen CTS Personas within the 10 clusters. As twelve profiles were the goal of the project, two of the clusters were the source of two roles each. Based on the tallies of the votes, the highest-scoring dozen roles, as shown below, were chosen to profile:

- Basic Scientist
- Biostatistician
- Clinical Research Center Administrator
- Clinical Research Coordinator
- Community-Engaged Researcher
- Data Analyst
- Developer
- K Scholar
- Librarian
- Patient Navigator
- Physician Scientist
- Research Administrator

### Step 2: Select Elements for a Well-defined Persona Profile

Persona profiles employed in UX design have several common elements, as confirmed by Usability.gov [2], the European Bioinformatics Institute [11], and UX Magazine [12], such as a photograph of the fictional, profiled person, a name, a biographical sketch, and listings of their motivations, goals, experience, knowledge, and pain points. The Personas team noted these elements and was also inspired by a similar profile layout produced by the Collections as Data project funded through the Institute of Museum and Library Services [13]. Personal attributes of a persona such as a photograph, name, and quote were deemed essential by the team, to reinforce that the Persona profiles represent real people working in CTS, with real motivations and needs. Though created from aggregated data, realistic persona qualities help make their needs real and urgent to the developer or other resource creator who makes tools to serve them.

**Fig. 1.**
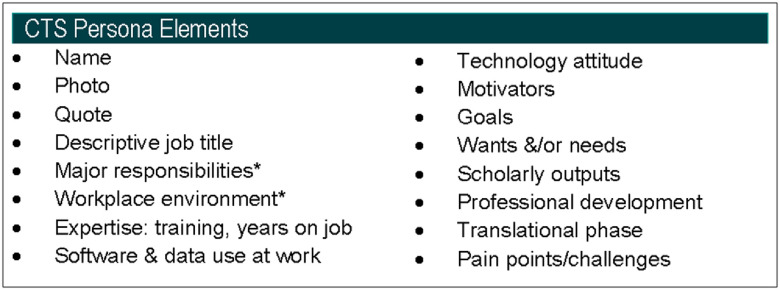
CTS Persona elements. Critical profile components used to profile the translational workforce through the CTS Personas project. *Incorporated in a biography section.

Building on these existing frameworks, the CTS Personas team added elements related to the scholarly output activities of members of the CTS workforce, as well as their continuing education goals and needs. Scholarly outputs in the form of peer-reviewed articles, conference presentations, and posters are key outputs for many in the translational workforce, with important implications for productivity, funding, and career success. Educational needs and motivators of the CTS workforce are of critical importance to CTSA Program hubs and CTSA Program organisational entities that serve them, such as the CD2H, the Trial Innovation Network, and the Center for Leading Innovation and Collaboration (CLIC). Knowledge of relevant scholarly activities and outputs, as well as continuing education needs of the profiled CTS roles, supports numerous initiatives within the ecosystem of clinical research support organisations. With these objectives in mind, the Personas team selected 16 elements to be included in the final, robust 1-page persona profiles (Fig. 1).

### Steps 3 and 4: Inform the Profiles from Literature Searches and Interviews

Searches for literature on the CTS workforce were conducted in PubMed and Scopus. PubMed was an invaluable tool for this research as a free and open archive of citations and publications (See Supplement 1, “Search Strategies”). References were shared with the project team through an EndNote library, and full-text articles were shared through a Google folder. Only around 60 articles were identified as directly informative for the CTS Personas project, demonstrating that workforce studies are an area meriting greater examination by the CTSA Program overall. The published literature was supplemented with internet resources and professional organisations’ websites such as NCURA and ACRP [14,15] for various roles, as well as job descriptions.

Advocates for leveraging best practices while creating persona profiles fall into two camps regarding the best way to dispel any assumptions or misconceptions that the persona creators may have about certain roles. Some, such as Cao [16], Natoli [17], and UX for the Masses Magazine [18], advocate role-playing or empathy exercises to uncover the key motivators for certain roles. Others, including the European Bioinformatics Institute [19] and UX Magazine [12], advocate interviews with exemplars of the roles as the best way to create persona profiles that are authentic and reliable. The CTS Personas team opted to conduct interviews to inform the profiles, with a goal of interviewing three people per position.

Northwestern led the interviews after completing review by the Institutional Review Board (IRB), resulting in the work being declared exempt. Interviews with employees of CTSA Program-funded translational sciences institutes across the majority of roles profiled were completed in Summer 2019. Given the time constraints of scheduling and completing interviews within the six-month project timeframe, three interviews per role were not always possible. The team averaged two interviews per role, and due to lack of time, no interviewees were identified for the Patient Navigator and Clinical Research Center Administrator roles. The Personas team was fortunate to locate a generous amount published resources on these two roles, which were used to complete the profiles in lieu of interviews [20]. The team agreed to showcase patient interactions with CTSA-supported resources through profiles as well, but opted to complete the patient profiles strictly from literature and internet research rather than interviews. The project’s IRB exempt determination was predicated on interviewees being members of the translational workforce. Interviews with patients in the translational landscape would have changed the project scope and timeframe considerably as involving human subjects research. As in the case of the Patient Navigator and Clinical Research Center Administrator, ample resources were found on patient/health care provider interactions to inform the two patient profiles [20].

Interviews were audio-recorded using encrypted BlueJeans conference sessions. The recordings were later transcribed by Scribie [21], an online transcription service that guarantees confidentiality and secure file transfer. Interviews were de-identified by the interviewer and analysed for data to populate the profiles. CTS Persona project interview questions were informed by sources such as the Interaction Design Foundation [22] and Usability.gov [23] and were designed to provide data to inform the persona profile elements listed under Step 2. A full list of the interview questions is provided as a supplement to this paper. (Supplement 2)

Data analysis was completed through a qualitative process. As the job descriptions and interview and literature data were gathered, quotes from each were added to lists on a Google doc with headings representing the Persona profile elements. Data points that were confirmed across the various sources of the job descriptions, literature, and interviews were included under headings such as responsibilities, software use, motivators, goals, pain points, and continuing education goals and needs. These commonly occurring data points were then incorporated into the final Persona profile layouts.

### Step 5: Construct the Profiles and User Guidebook

**Fig. 2.**
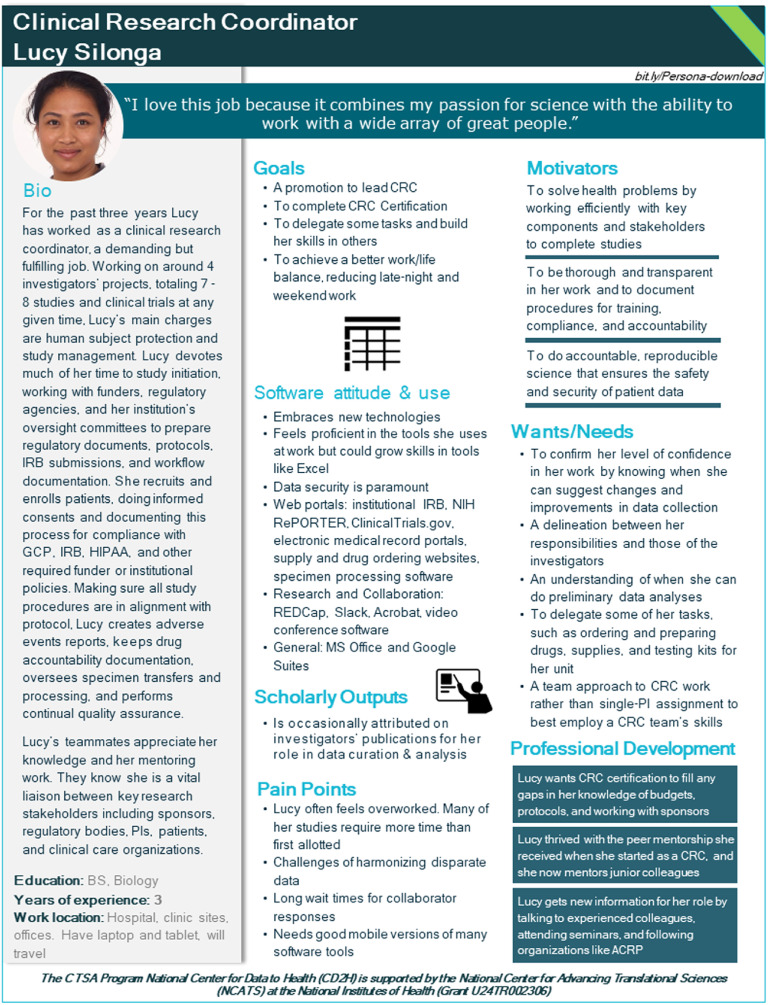
Sample Persona, here for a clinical research coordinator.

One-page profile documents were written once all data were compiled from the literature search and de-identified interview transcripts. The iterative process benefited from the participation and input of all the CTS Personas team members through regular biweekly calls. Feedback was collected on early versions of the profiles, which enabled the generation of succinct, accessible, and intuitively designed one-page profiles (Fig. 2).

The website This Person Does Not Exist [24] provides artificial intelligence-generated images of human faces that are based on no real person and was used as the source of all fourteen of the persona profiles’ headshots. Team members associated one image with each role and gave each persona a fictional name.

**Fig. 3.**
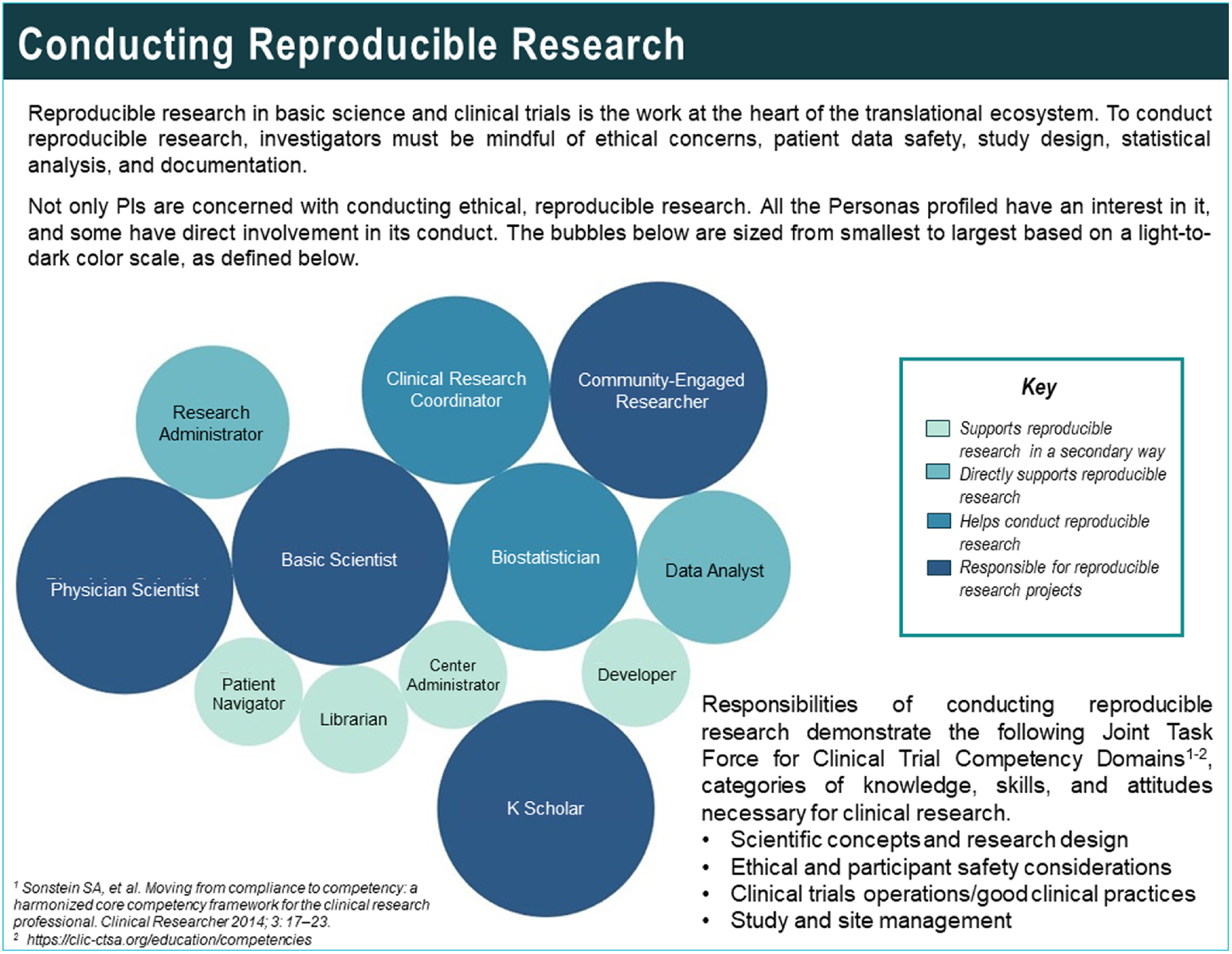
An example of a visualisation from a secondary data analysis.

**Fig. 4.**
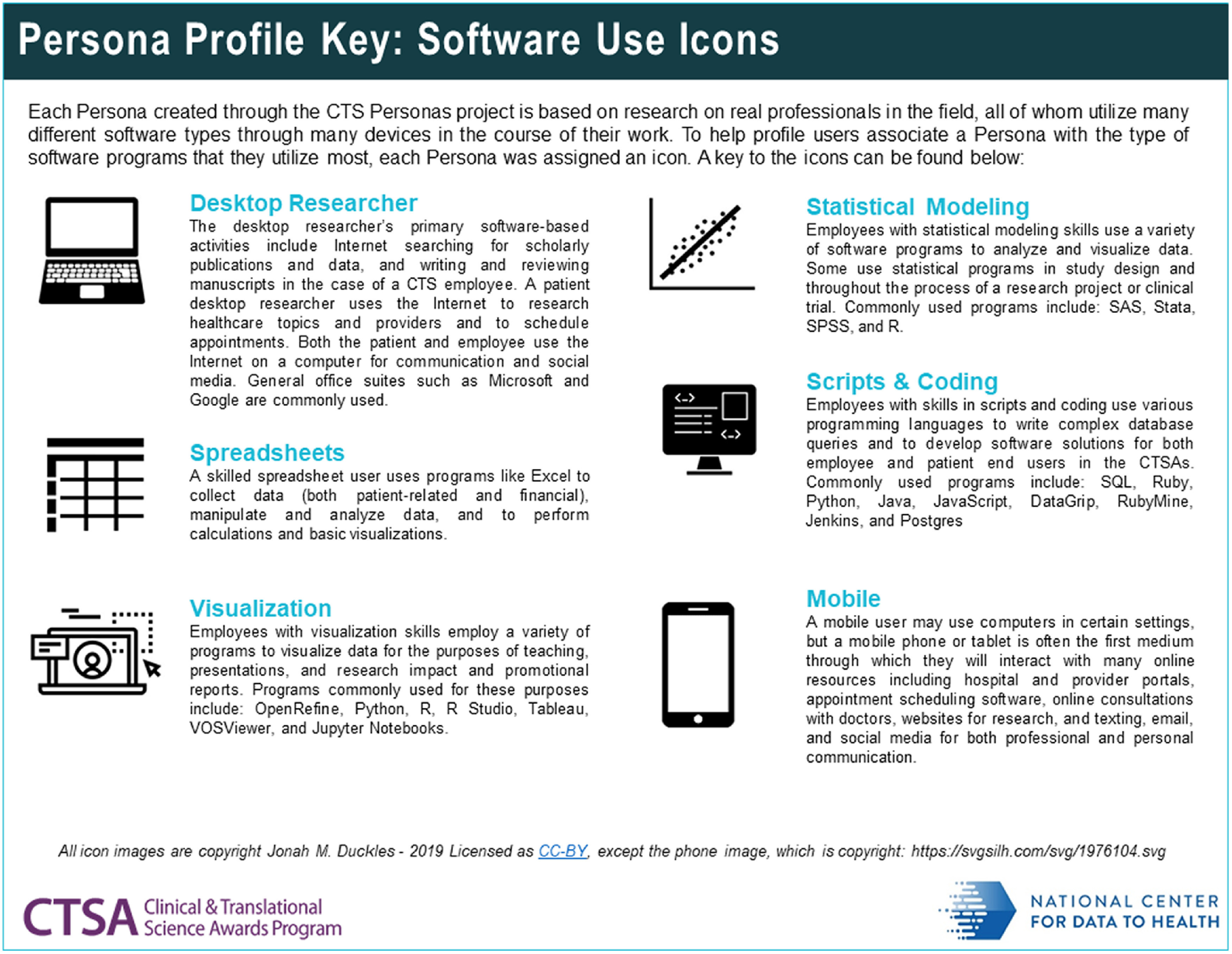
Persona software icons used to indicate software skill level.

**Fig. 5.**
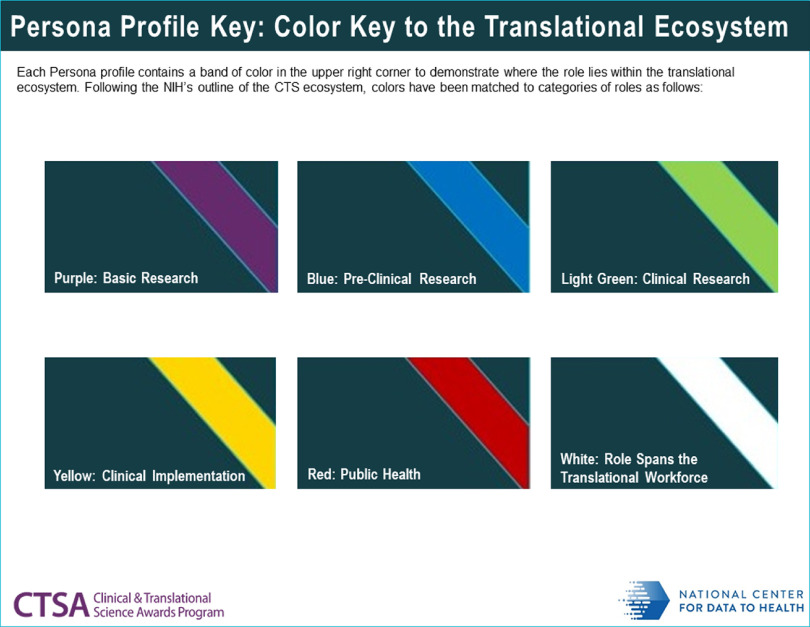
Colour coding used to orient the reader to the translational phase(s) for a given persona.

For certain elements of the profiles, more detailed information was generated than could be provided through the one-page profiles alone. To address this need, the project team created a user guidebook [20]. The guidebook contains secondary analyses and visualisations elucidating the supplementary information behind some of the personas elements (Fig. 3). Also included are explanations of the software icons that are included in each profile, which serve as a visual demarcation of the individual persona’s level of skill with certain types of software and technology (Fig. 4). The bands of colour at the top right corner of each profile denote where the persona’s position is located on the spectrum of translational science and are highlighted in the Personas guidebook, as well (Fig. 5). Keys to the elements of both the employee and patient profiles, as well as a bibliography and credits, are also included.

**Fig. 6.**
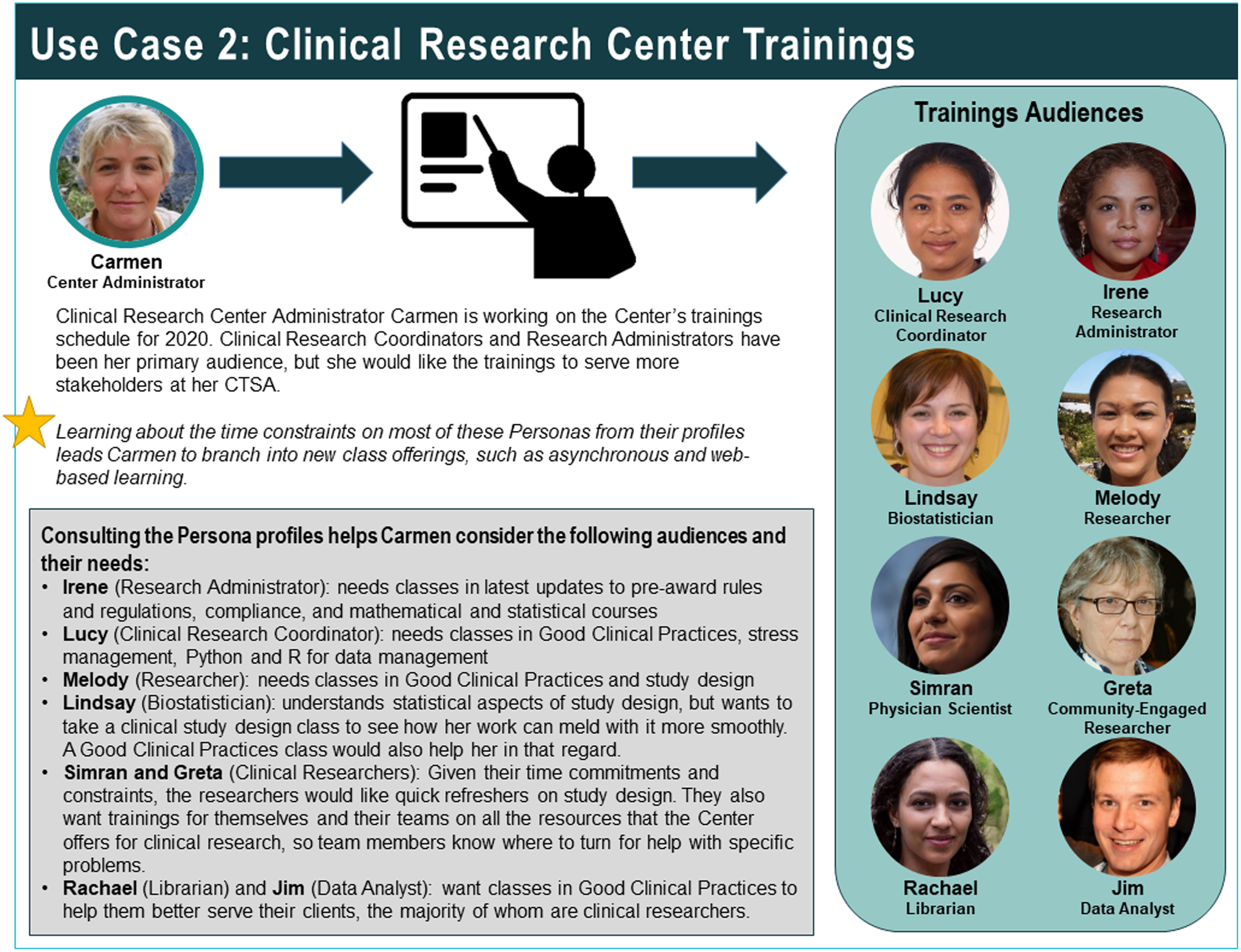
Sample use case from the CTS Personas guidebook.

A special feature of the guidebook is three sample use cases demonstrating how the persona profiles can be employed to improve products and processes at CTSA institutions. Through brief examinations of scenarios involving (1) building an end-to-end patient platform, (2) developing clinical research centre trainings (Fig. 6), or (3) building a software development team, it was demonstrated how nearly all of the CTS roles and patients profiled would have some bearing on the development of these tools and resources.

## CTS Personas Launch and Engagement

CTS Personas officially launched on 9 September, 2019. A GitHub Pages site [25] was created from the main GitHub site containing the completed profiles, additional information about the employees’ software use, the user guidebook, the sample use cases as separate documents, a frequently asked questions page, and a feedback page. The profiles and user guidebook are downloadable both from the project page and from Northwestern University Feinberg School of Medicine’s institutional repository, DigitalHub [26]. A series of trading cards for each of the personas was also designed and printed for dissemination at CTSA Program events. Each card highlights a persona, some key attributes of the role, and provides URLs back to the individual persona and the GitHub project site.

Announcements of the availability of the persona profiles were sent through the communication channels of the CD2H and the CLIC in the CTSAs, listservs of various research and professional organisations, and FORCE11 [27]. Additional engagement avenues include an introductory webcast video on the GitHub page and an onboarding webinar presented on 13 November, 2019 [28].

## Conclusion and Lessons Learned

One of the lessons learned through creating fourteen translational workforce and patient profiles was the value of allocating sufficient time and resources to a personas project. This is particularly true if conducting interviews to inform the profiles. Due to the time constraints of many working in CTS, it can be difficult to obtain interviews, and it is often best to have the flexibility to conduct them at a time that accommodates the interviewees’ best available time slots. Time limitations on the CTS Personas project precluded obtaining interviews for every role profiled, with ten of the twelve CTS employee roles ultimately being informed by interviews.

Empathy is a key component of any project to create personas, as the goal is to understand a position or career from the point of view of those living it every day. To effectively obtain information about motivations and pain points that people experience in their jobs, interviewers must demonstrate empathy, understanding, and trustworthiness to their interviewees. Working with an interviewer skilled in social-cultural research methods, or completing a class or workshop in unbiased and empathetic interview techniques, can be a helpful first step in conducting effective interviews.

CTS Personas has been a timely and valuable project for the CD2H and CTSA sites, receiving acknowledgement through social media and listservs and multiple downloads of the project materials within the first week of its launch. Tool-sharing, informatics fluency, and collaboration amongst those employed in CTS can be informed, and tools to meet these needs can be more effectively designed, through examination of the real-life motivations, goals, needs, wants, and pain points of the dozen roles profiled. But more than that, the process of persona profile creation has been instructive and fortifying to the CTS Personas team itself. From the evidence provided by interviews and from reviewing the literature on various workforce roles, each member of the team has come away from the project with a greater understanding of and appreciation for their colleagues in CTS, from those working tirelessly to activate trials to those designing the databases that will enable analysis and aggregation of data. The CTS Personas team encourages the CTSA community to build on our methodology and efforts and continue the work of creating persona profiles that reflect the work and achievements of those employed in the CTSAs.

## Supplementary material

For supplementary material accompanying this paper visit https://doi.org/10.1017/cts.2020.2. click here to view supplementary material
