# Supplementary material for: Personas for the translational workforce
Source: J Clin Transl Sci. 2020 Jan 10;4(4):286–93. doi: 10.1017/cts.2020.2 (PMC7681142; doi:10.1017/cts.2020.2)
Supplement: Supplementary file 1 [file S2059866120000023sup.zip › S2059866120000023sup001.docx]

**Personas for the Translational Workforce**

**Supplement 2: Interview Questions**

1.      What is your job title?

2.      Please provide a little background on your job.

3.      What projects might you work on during a typical day?

4.      How long have you been working in this capacity?

5.      Did you train for this specific job? What was your educational path?

6.      Please provide some information about your field of work and your role in it.

7.      Describe a typical work day.

8.      How and where do you do a certain task? How long does it take? What do you do next?

9.      What activities take up most of your time (Follow up: From 0 - 10, what is the frequency with which this task occurs in a given week?)

10.   What activities are most important to your success (as you define success)?

11.   Of the things you do during a typical workday, are any of those processes or tasks mandated by your industry/hospital/lab/office/etc.?

12.   What was your most exciting project?

13.   What was your most challenging project?

14.   What about your work environment either helps or hinders you in these tasks?

15.   What software and data tools do you use on a daily basis?

16.   What did you feel, what went on in your head, the last time (insert software tool from #15) didn’t work?

17.   How would you describe your relationship with technology? (E.g., proficient in many, some, or few applications. Are these general technology applications or role-specific? Does your proficiency level differ between general applications and work-related ones?)

18.   What do you like most about your work? What would you change?

19.   What goals are you working toward at the moment?

20.   Are these your goals or team goals? If these are different, please explain how.

21.   What blocks you from completing your goals?

22.   After a typical day, what about your job (if anything) is still on your mind?

23.   What professional groups, blogs, publications, and social media networks do you pay most attention to?

24.   What are your most common scholarly outputs? (E.g., articles, conference materials, classes, etc.)

25.   Does your department support you in taking continuing education or furthering your training in some way? If so, would you like to share which classes you are taking? Are you working towards a goal in your continuing education?
