# Supplementary material for: Personas for the translational workforce
Source: J Clin Transl Sci. 2020 Jan 10;4(4):286–93. doi: 10.1017/cts.2020.2 (PMC7681142; doi:10.1017/cts.2020.2)
Supplement: Supplementary file 1 [file S2059866120000023sup.zip › S2059866120000023sup002.docx]

**Personas for the Translational Workforce**

**Supplement 1: PubMed and Scopus Search Strategies**

**PubMed:**

("Translational Medical Research"[Mesh] OR Translational science[tiab] OR Translational research[tiab] OR CTSA[tiab] OR CTSI[tiab]) AND (Center[tiab] OR Centers[tiab] OR Centre[tiab] OR Centres[tiab] OR Institute*[tiab] OR Award[tiab]) AND (job[tiab] OR jobs[tiab] OR position[tiab] OR positions[tiab] OR role[tiab] OR roles[tiab] OR personas[tiab])

**Scopus:**

Keyword searching on the topics (translational science centers AND roles), organized by citation count to locate highly cited articles
